# DISCRIMINATION AND MENTAL HEALTH AMONG OLDER ADULTS: THE CONFOUNDING ROLE OF PERSONALITY

**DOI:** 10.1093/geroni/igae098.2670

**Published:** 2024-12-31

**Authors:** Morgan Stangl, Man Guo, Yi Wang

**Affiliations:** University of Iowa, Iowa City, Iowa, United States; University of Iowa, Iowa City, Iowa, United States; University of Iowa, Iowa City, Iowa, United States

## Abstract

Existing literature shows that the perception of discrimination is related to poorer mental health. Personality may confound this association by shaping an individual’s coping style, social support seeking, and emotional regulation in the face of adversity. However, such a confounding role of personality hasn’t been well studied, and it’s unclear whether different personality traits have similar effects. Using data from middle-aged and older adults (aged 50+) in the 2018 Health and Retirement Study Psychosocial Leave-Behind Questionnaire (N = 5,529), this research delves deeper into the potential moderating role of two personality traits (neuroticism and extraversion) in the associations between perceived discrimination and mental health outcomes (depression and anxiety). Findings from regression analyses revealed that increased perceived discrimination was consistently associated with higher levels of anxiety and depression. While such associations did not vary based on individuals’ levels of neuroticism, the associations were weaker among individuals with a higher level of extraversion. This study revealed that the two personality traits, neuroticism and extraversion, have different effects on the relationship between perceived discrimination and mental health. Extroverted individuals may perceive and cope with discrimination differently with more adaptive coping mechanisms, such as seeking social support and engaging in problem-solving behaviors, which may buffer against the negative effects of discrimination on mental health. Further studies of the underlying mechanisms are needed. This can inform the customization of mental health interventions to align with individuals’ personality profiles for better-tailored and targeted support that effectively promotes their resilience in the face of discrimination.

